# Hybridization of Taguchi and Genetic Algorithm to minimize iteration for optimization of solution

**DOI:** 10.1016/j.mex.2019.01.002

**Published:** 2019-01-15

**Authors:** P.A. Vaghela, J.M. Prajapati

**Affiliations:** a Department of Mechanical Engineering, R C Technical Institute, Gujarat Technological University, Ahmedabad, Gujarat, India; b Department of Mechanical Engineering, Faculty of Technology and Engineering, The Maharaja Sayajirao University of Baroda, Vadodara, Gujarat, India

**Keywords:** Genetic algorithm, Taguchi, Optimization, Spur gear, Initial population

## Abstract

This paper describes a novel hybrid approach of Taguchi and Genetic Algorithm to minimize number of iteration for optimization of a solution of the problem. A Genetic algorithm is used for global optimization. In GA initial population is selected randomly. Taguchi method gives a uniform combination of variables for the given search area. Hence, instead of selecting the initial populations by random search select the initial population by Taguchi design techniques. It will reduce the number of iteration to obtain a solution. This is explained with illustration.

• It can be used for selecting initial population in an organized manner rather than random selection.
• It can reduce the number of iterations.
• It can be applicable to all optimization problems where Genetic Algorithm is used.

## Graphical abstract

**Figure:**
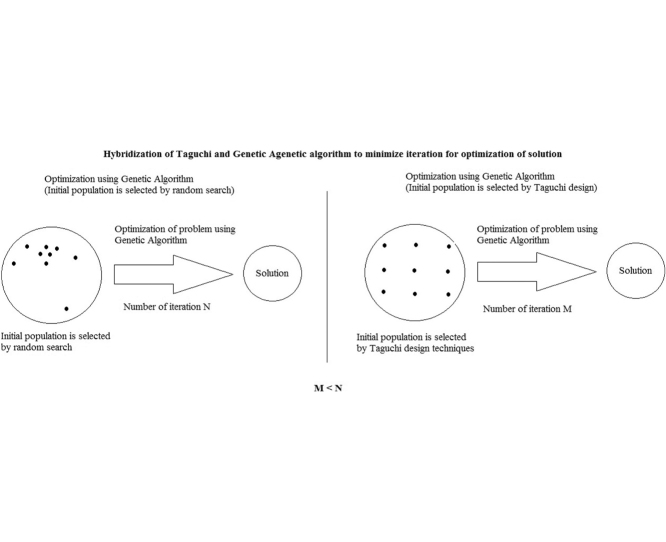

## Nomenclature

*α_Fen_*: Load angle
*α_n_*: Normal pressure angle
*α_c_*: Coast side pressure angle
*α_d_*: Drive side pressure angle
*ε*: Contact ratio
*σ*F: Bending stress
b: Effective face width
*S_Fn_*: Critical section thickness
*h_Fe_*: bending arm height
*F_t_*: Tangential load
*i*: Speed ratio
*k*: Asymmetry factor
*S_td_*: Tip thickness at addendum circle on the drive side
*S_tc_*: Tip thickness at addendum circle on the coast side
*S_t_*: Tip thickness at addendum circle
*m*: Module
*m_n_*: Normal module
*T_1,_ T_2_*: Torque on the pinion (1), gear (2)
*r*: Any radius
*r_p_*: Pitch circle radius
*r_bc_*: Base circle radius at coast side
*r_HPSTC_*: Radius at HPSTC
*r_t1_/r_t2_*: Addendum circle radius on pinion (1), Gear (2)
*r_b1_/r_b2_*: Base circle radius on pinion (1), Gear (2)
*C*: Centre distance
*θ_Fnd_*: Angle at base circle radius coast side w.r.t y-axis on the drive side
*θ_Fnc_*: Angle at base circle radius coast side w.r.t y-axis on the coast side
*θ_HPSTC_*: Angle at HPSTC radius w.r.t y-axis
*z_p_*: Total tooth on the pinion
*z_g_*: Total tooth on the gear
*Y_F_*: Tooth form factor
*Y_B_*: Helix angle factor
*Y_S_*: Stress correction factor
*ρ_F_*: Normal fillet radius at the root of a tooth
HPSTC: Highest point of single tooth in contact

**Specifications Table**

**Table:** 

Subject Area:	• Engineering • Mathematics
More specific subject area:	Optimization of problem using Genetic Algorithm.
Method name:	Selection of initial population using Taguchi for Genetic Algorithm.
Name and reference of original method:	This study combines two techniques, Taguchi method and Genetic Algorithm. Taguchi method is used for selecting initial population in an organized manner rather than random selection.
	John Holland introduced genetic algorithms in 1960 based on the concept of Darwin’s theory of evolution; afterwards, his student Goldberg extended GA in 1989.
	Taguchi methods are statistical methods, or sometimes called robust design methods, developed by Genichi Taguchi.
Resource availability:	Taguchi Method.
	Genetic Algorithm.

### Method details

Various methods are available to optimize a solution. The genetic algorithm is one of them and used for global optimization. GA is a work based on natural selection and fittest to survival. In GA initial population is required and it was selected by random search. GA gives solution after some iteration based on the complexity of problem [1].

Taguchi design technique gives more variables with fewer experiments using orthogonal array method. It also gives a good combination of variables within the given search area [2].

Various studies are available in the literature for hybridization of Taguchi method and Genetic Algorithm. In all hybridization, focus on a selection of initial population by Taguchi method is untouched. In this paper, instead of selecting initial population by random search, select the initial population by Taguchi design techniques. It will reduce the number of iteration to obtain a solution. This is explained with illustration.

### Problem/Illustration

Gears are a very important part in power transmission. Durability and consistency in power transmission is the main focus in gear design. To improve higher durability in power transmission, current focus of in industry is on the failure occurring at a critical section of the tooth [3,4].

**Fig. 1:**
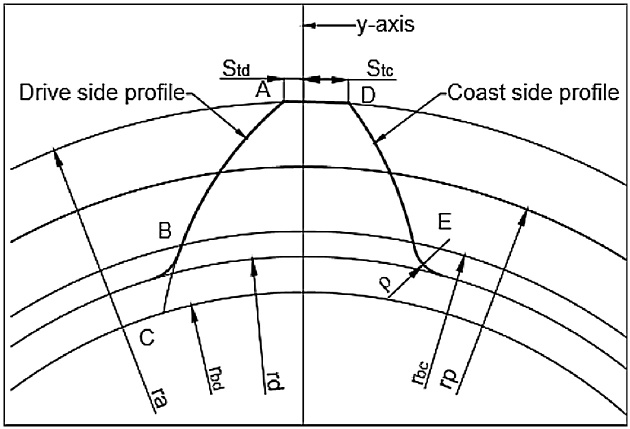
Asymmetric involute spur gear tooth profile.

Failure at critical section of the tooth can be reduced by minimizing bending stress at critical section of the tooth. Higher pressure angle on the drive side is desirable to minimize bending stress at the root of a tooth [5,6]. It will change geometrical shape of standard symmetric gear tooth. Due to different pressure angle on both sides, standard normal gear becomes asymmetric gear (Fig. 1)[5,6].

#### Parameters

For illustration, the parameters are given in Table 1 [7,8].

**Table 1:** Parameters.

	Parameters	Value	Unit
1	Pressure angle on coast side	20°	Degree
2	Pressure angle on drive side	20°–38°	Degree
3	Number of teeth on gear and pinion	25 and 25	–
4	Module	4	mm
5	Power	18	KW
6	Rotation	1600	RPM

#### Objective function/fittness function

In this illustration, the main aim is to minimize bending stress at the critical section. Objective function/fitness function has been considered from reference [8] to calculate fitness value in the Genetic Algorithm.

Objective function/fitness function to minimize bending stress at the critical section with constraint:

Nominal tooth root stress $\sigma_{F0}$

(When a load acts at HPSTC) $\sigma_{F0(\min)}=f\left(\frac{F_{t}}{b\cdot m_{n}}Y_{s}\cdot Y_{F}\cdot Y_{\beta }\right)$

Constraints are: $\alpha_{d}>\alpha_{c}$, ε≥1.1, *S_t_* ≥ *0.25*m

$$Y_{F}=\frac{6\cdot \left(h_{Fe}/m_{n}\right)\cos\alpha_{Fen}}{{\left(S_{Fn}/m_{n}\right)}^{2}\cos\alpha_{n}}$$

$$\alpha_{Fen}=\tan\left(\cos^{-1}\left(\frac{r_{p}}{r}\cdot \cos\alpha \right)\right)-\frac{\pi }{2\cdot z}-\tan\alpha +\alpha$$

$$S_{Fn}=r_{bc}.\sin\theta_{Fnd}+r_{bc}.\sin\theta_{Fnc}$$

Where,

$$\theta_{Fnd}=\frac{\pi }{2.z}+\tan\alpha_{d}-\alpha_{d}-\left(\tan\left(\cos^{-1}\left(\frac{r_{p}}{r_{bc}}.\cos\alpha_{d}\right)\right)-\left(\cos^{-1}\left(\frac{r_{p}}{r_{bc}}.\cos\alpha_{d}\right)\right)\right)$$

$$\theta_{Fnc}=\frac{\pi }{2.z}+\tan\alpha_{c}-\alpha_{c}-\left(\tan\left(\cos^{-1}\left(\frac{r_{p}}{r_{bc}}.\cos\alpha_{c}\right)\right)-\left(\cos^{-1}\left(\frac{r_{p}}{r_{bc}}.\cos\alpha_{c}\right)\right)\right)$$

**Fig. 2:**
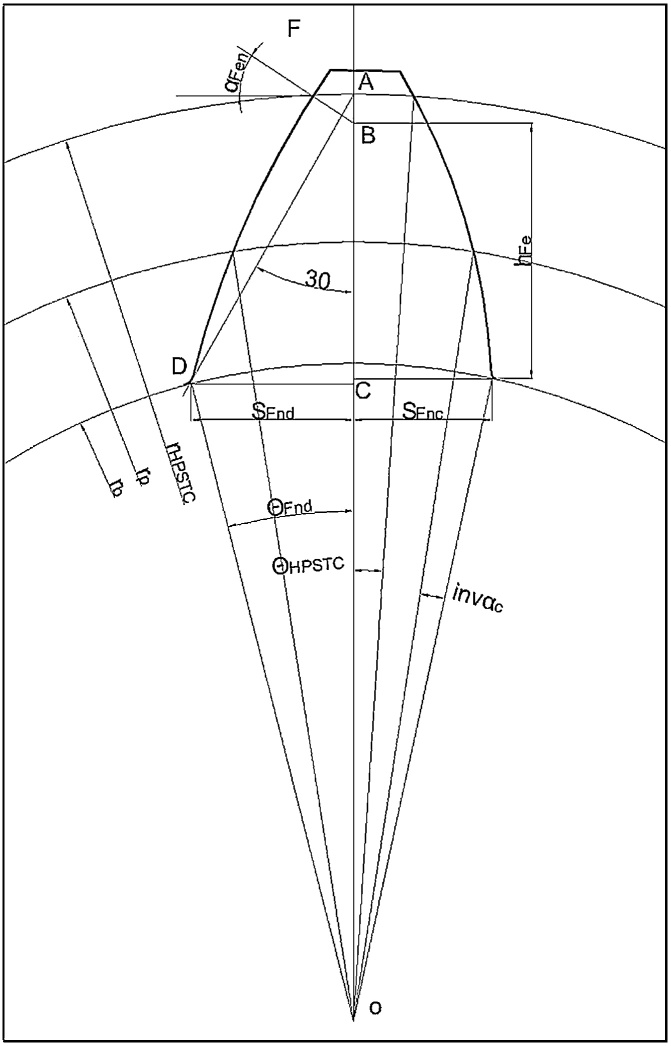
Representation of bending stress equation parameters.

Fig. 2 shows the asymmetric involute spur gear tooth with bending stress equation parameters.

$$h_{Fe}=r_{HPSTC}\cdot \cos\theta_{HPSTC}-\left(r_{bc}\cos\theta_{Fnd}+r_{HPSTC}\cdot \sin\theta_{HPSTC}\cdot \tan\alpha_{Fen}\right)$$

Where,

$$\theta_{HPSTC}=\frac{\pi }{2.z}+\tan\alpha -\alpha -\left(\tan\left(\cos^{-1}\left(\frac{r_{p}}{r_{HPSTC}}.\cos\alpha \right)\right)-\left(\cos^{-1}\left(\frac{r_{p}}{r_{HPSTC}}.\cos\alpha \right)\right)\right)$$

$$\theta_{Fnd}=\frac{\pi }{2.z}+\tan\alpha -\alpha -\left(\tan\left(\cos^{-1}\left(\frac{r_{p}}{r_{bc}}.\cos\alpha \right)\right)-\left(\cos^{-1}\left(\frac{r_{p}}{r_{bc}}.\cos\alpha \right)\right)\right)$$

$$Y_{\beta }=1-\varepsilon_{\beta }\frac{\beta }{120}$$

$$Y_{S}=\left(1.2+0.13L\right)q_{s}^{a}$$

$$L=S_{Fn}/h_{Fe},q_{s}=S_{Fn}/\left(2\rho_{F}\right),a={\left[1.21+2.3/L\right]}^{-1}$$

ρ_F= 0.3 m_

### Optimization using GA (Initial population selected by random search)

#### GA algorithm [1]

**Table:** 

Step 1	Generate initial value of the genes for chromosome	
Step 2	Calculates fitness of objective function	
	Step 2.1	Start
	Step 2.2	Input m, *z_p_*, *i*, *α*_c_ and *α_d_*
	Step 2.3	Calculate variables: h_Fe_, S_Fn_, Y_F_, Y_S_, Y_β_, $\rho_{F}$
	Step 2.4	Calculate bending stress at root of tooth
	Step 2.5	End
Step 3	Selection (Roulette wheel)	
	Step 3.1	Start
	Step 3.2	Find f_obj for selected population
	Step 3.3	Find $F_{i}=1/\left(1+F\_obj_{i}\right)$
	Step 3.4	$\text{Calculate Probability}P_{i}=F_{i}/\left(\sum_{j=1}^{n}F_{j}\right)$
	Step 3.5	Calculate $\text{Cumulative probability}{\text{C}}_{\text{i}}=\left(\sum_{i=1}^{N=5}P_{i}\right)$
	Step 3.6	Generate random numbers R_i_ in the range 0-1
	Step 3.7	If (R[i]>C[i] & R[i] <C [i+1]) then select chromosome C [i+1]
	Step 3.8	Repeat for all population
	Step 3.9	End
Step 4	Crossover (**one-point crossover)**	
	Step 4.1	Start
	Step 4.2	select crossover_rate (ρc) parameters
	Step 4.3	generate a random number R[k] as the number of population N.
	Step 4.4	if R[k]< ρc then select kth no. chromosome selected for crossover until N population
	Step 4.5	generating random numbers equal to no. of chromosome select in step 4, between 1 to (length of Chromosome – 1).
	Step 4.6	Chromosome will be cut at a crossover point and its gens will be interchanged
	Step 4.7	End
Step 5	Mutation	
	Step 5.1	Start
	Step 5.2	Select mutation rate parameter
	Step 5.3	calculates the total length of gen in the population. Total length of gen is total_gen = number_of_gen_in_Chromosome *number of population
	Step 5.4	calculate number of mutations = mutation rate * total_gen
	Step 5.5	generating gene equal to the number of mutations as per constraints
	Step 5.6	mutated genes at a mutation point are replaced by new gens
	Step 5.7	obtain new chromosome
	Step 5.8	End
Step 6	if bending stress = 45.60 Mpa [Obtain from reference 7, 8] --- > yes go to step 7 otherwise go to step 2	
Step 7	Calculate the number of iteration	
Step 8	End	

#### Initial population

9 chromosomes have been created using random search method. Parameters such as drive side pressure angle (*α_d_)*, Contact ratio (*ε)* and tip thickness (*S_t_*)are considered as a gene for chromosomes.

As the pressure angle on the drive side (as compared to the coast side) increases, the bending stress decreases at root of the tooth [5,6]. But contact ratio (ε) and tip thickness (S_t_) are constraints for the same. Gear design standard procedures recommend that, the contact ratio should be higher or equal to 1.1 [3,4]. Below this contact ratio, tooth loading period increases and it is an undesirable condition for cyclic loading.

$$\varepsilon =\frac{\sqrt{r_{t1}^{2}-r_{b1}^{2}}+\sqrt{r_{t2}^{2}-r_{b2}^{2}}-C\cdot \sin\alpha }{p\cdot \cos\alpha }$$

Gear design standard procedures recommend that, the tip thickness should be ≥ 0.4*m* for hardened gears. In exceptional cases, tip thickness decreases to 0.25*m* [3,4]. Below this tip thickness it becomes more pointed.

**Tip thickness for symmetric spur gear tooth**

$$S_{t}=2\cdot r_{t}\cdot \theta_{t}$$

$$\theta_{t}=\frac{\pi }{2.z}+\tan\alpha -\alpha -\left(\tan\left(\cos^{-1}\left(\frac{r_{p}}{r_{t}}.\cos\alpha \right)\right)-\left(\cos^{-1}\left(\frac{r_{p}}{r_{t}}.\cos\alpha \right)\right)\right)$$

**Tip thickness of asymmetric spur gear tooth**

$$S_{t}=r_{t}.\theta_{td}+r_{t}.\theta_{td}$$

$$\theta_{td}=\frac{\pi }{2.z}+\tan\alpha_{d}-\alpha_{d}-\left(\tan\left(\cos^{-1}\left(\frac{r_{p}}{r_{t}}.\cos\alpha_{d}\right)\right)-\left(\cos^{-1}\left(\frac{r_{p}}{r_{t}}.\cos\alpha_{d}\right)\right)\right)$$

$$\theta_{tc}=\frac{\pi }{2.z}+\tan\alpha_{c}-\alpha_{c}-\left(\tan\left(\cos^{-1}\left(\frac{r_{p}}{r_{t}}.\cos\alpha_{c}\right)\right)-\left(\cos^{-1}\left(\frac{r_{p}}{r_{t}}.\cos\alpha_{c}\right)\right)\right)$$

Based on the above equations and parameter given in Table 1, the range of tip thickness is between 1.26 to 2.88, the range of contact ratio is between 1.20 to 1.61 and range of drive side pressure angle is between 20° to 38°.

Created initial populations selected by random search are:

Chromosome [1] = [α_d_; ε; *S_t_*] = [20; 1.50; 2.88]

Chromosome [2] = [α_d_; ε; *S_t_*] = [22; 1.60; 1.90]

Chromosome [3] = [α_d_; ε; *S_t_*] = [20; 1.61; 2.60]

Chromosome [4] = [α_d_; ε; *S_t_*] = [30; 1.20; 1.30]

Chromosome [5] = [α_d_; ε; *S_t_*] = [35; 1.55; 1.50]

Chromosome [6] = [α_d_; ε; *S_t_*] = [30; 1.61; 2.16]

Chromosome [7] = [α_d_; ε; *S_t_*] = [38; 1.25; 1.31]

Chromosome [8] = [α_d_; ε; *S_t_*] = [38; 1.60; 2.80]

Chromosome [9] = [α_d_; ε; *S_t_*] = [35; 1.61; 1.80]

#### Solutions

As per reference [7], it is found that optimized or minimized bending stress for given parameters is 45.60 Mpa. The algorithm is presented to solve a problem using GA in section 3.1. Using GA, it is found that 15 iterations are required to obtain optimum bending stress when initial population is selected by random search.

### Optimization using GA (Initial population selected by the Taguchi method)

#### The algorithm to select initial population for GA using Taguchi method [2]

Step 1: Select the number of parameters

Drive side pressure angle (*α_d_)*, Contact ratio (*ε)* and tip thickness (*S_t_*) parameters have been selected.

Step 2: Select the number of levels

Three levels have been selected

Step 3: Select orthogonal array as per a number parameters and levels using standard table

Standard table is given in Table 2. It gives orthogonal array based on number parameters and number of levels. L9 orthogonal array selected based on number of parameters and number of levels.

**Table 2:** Standard table.

	Parameters											
L E V E L S		2	3	4	5	6	7	8	9	10	11	12
	2	L4	L4	L8	L8	L8	L8	L12	L12	L12	L12	L16
	3	L9	L9	L9	L18	L18	L18	L18	L27	L27	L27	L27
	4	L16	L16	L16	L16	L32	L32	L32	L32	L32		
	5	L25	L25	L25	L25	L25	L50	L50	L50	L50	L50	L50

Step 4: Create the orthogonal array

Standard L9 orthogonal array is given in Table 3, which gives the best combinations among a number of runs.

**Table 3:** Standard L9 orthogonal array.

		Experiment Number								
		1	2	3	4	5	6	7	8	9
LEVEL	A	1	1	1	2	2	2	3	3	3
	B	1	2	3	1	2	3	1	2	3
	C	1	2	3	2	3	1	3	1	2

In present study, drive side pressure angle (α_d_), Contact ratio (ε) and tip thickness (S_t_) are considered as a performance parameter. Developed L9 orthogonal array is given in Table 4.

**Table 4:** Developed l9 orthogonal array.

		Experiment Number								
		1	2	3	4	5	6	7	8	9
LEVEL	A	20	20	20	29	29	29	38	38	38
	B	1.20	1.40	1.61	1.20	1.40	1.61	1.20	1.40	1.61
	C	1.26	2.07	2.88	2.07	2.88	1.26	2.88	1.26	2.07

Step 5: create chromosomes based on developed orthogonal array

Chromosome [1] = [α_d_; ε; *S_t_*] = [20; 1.20; 1.26]

Chromosome [2] = [α_d_; ε; *S_t_*] = [20; 1.40; 2.07]

Chromosome [3] = [α_d_; ε; *S_t_*] = [20; 1.61; 2.88]

Chromosome [4] = [α_d_; ε; *S_t_*] = [29; 1.20; 2.07]

Chromosome [5] = [α_d_; ε; *S_t_*] = [29; 1.40; 2.88]

Chromosome [6] = [α_d_; ε; *S_t_*] = [29; 1.61; 1.26]

Chromosome [7] = [α_d_; ε; *S_t_*] = [38; 1.20; 2.88]

Chromosome [8] = [α_d_; ε; *S_t_*] = [38; 1.40; 1.26]

Chromosome [9] = [α_d_; ε; *S_t_*] = [38; 1.61; 2.07]

#### Initial population for GA using Taguchi method

An initial population has been developed using Taguchi methods. This will help in achieving the best combinations. A population has been given in Section 4.1- step 5.

#### Solutions

As per reference [7], it is found that optimized or minimized bending stress for given parameters is 45.60 Mpa. The algorithm is presented to solve a problem using GA in Section 3.1 and 4.1. Using GA along with Taguchi, it is found that 10 iterations are required to obtain optimum bending stress.

### Results and discussion

From the illustration presented in the previous section, number of iterations are 15 when initial population selected by random search and number of iterations are 10 when initial population is selected using Taguchi method. Comparison of number of iterations obtained from GA, when initial population is selected by random search and initial population is selected by Taguchi method is given in Table 5.

**Table 5:** Result table.

	Result table		
	Method	No. of Iteration	Bending stress (MPa)
1	Solution using GA	15	45.60
2	Solution using GA by selecting an initial population with help of Taguchi method	10	45.60

### Conclusion

It is observed that a number of iterations to solve a problem is reduced, when the Taguchi method is used to select the initial population instead of random search.
